# Salvage cytoreductive surgery for patients with recurrent endometrial cancer: a retrospective study

**DOI:** 10.1186/1471-2407-14-135

**Published:** 2014-02-26

**Authors:** Yulan Ren, Boer Shan, Daren Shi, Huaying Wang

**Affiliations:** 1 Department of Gynecologic Oncology, Fudan University Shanghai Cancer Center, Shanghai 200032, China; 2 Department of Oncology, Shanghai Medical College, Fudan University, Shanghai 200032, China; 3 Department of Pathology, Fudan University Shanghai Cancer Center, Shanghai 200032, China

**Keywords:** Endometrial cancer, Recurrence, Cytoreductive surgery, Prognosis

## Abstract

**Background:**

Salvage cytoreductive surgery (SCR) has been shown to improve the survival of cancer patients. This study aimed to determine the survival benefits of SCR for recurrent endometrial cancer in Chinese population.

**Methods:**

Between January 1995 and May 2012, 75 Chinese patients with recurrent endometrial cancer undergoing SCR were retrospectively analyzed.

**Results:**

43 patients (57.3%) had R0 (no visible disease), 15 patients (20.0%) had R1 (residual disease ≤1 cm), and 17 (22.7%) had R2 (residual disease >1 cm) Resection. 35 patients (46.7%) had single, and 40 (53.3%) had multiple sites of recurrence. The median survival time was 18 months, and 5-year overall survival (OS) rate were 42.0%. Multivariate analysis showed that residual disease ≤1 cm and high histology grade were significantly associated with a better OS. The size of the largest recurrent tumors (≤6 cm), solitary recurrent tumor, and age at recurrence (≤56 years old) were associated with optimal SCR.

**Conclusion:**

Optimal SCR and high histology grade are associated with prolonged overall survival for patients with recurrent endometrial cancer. Patients with young age, tumor size < 6 cm, and solitary recurrent tumor are more likely to benefit from optimal cytoreductive surgery.

## Background

Endometrial carcinoma is a common gynecological cancer and the majority of patients present at an early stage with a good long-term prognosis. However, about 13% of patients with endometrial cancer develop recurrent disease, and they have a very poor outcome with a mortality of about 25% [1,2].

Treatment options for recurrent endometrial cancer vary according to the distribution of recurrent disease. Chemotherapy is often recommended for patients with distant or widely metastatic recurrences, while radiotherapy is recommended for patients with small, isolated pelvic recurrences who have not received radiation [3,4]. For surgery, pelvic exenteration is recommended for the treatment of a localized central pelvic recurrence refractory to radiation therapy [5,6], while the benefit of salvage cytoreductive surgery (SCR) in patients with recurrent endometrial cancer is not confirmed. Several recent reports reported that selected patients with resectable recurrent endometrial cancer could benefit from complete SCR [7-10]. Therefore, the surgical indications and selection criteria are urgently needed to minimize the complications and mortality associated with surgery.

In this retrospective study, we evaluated the survival benefit and the safety of SCR for Chinese patients with recurrent endometrial cancer, and tried to define the selection indications for SCR.

## Methods

### Ethics statement

The study was approved by Ethics Committee of Fudan University. All patients who participated in the study signed informed consent forms.

### Study design

All patients with recurrent endometrial cancer undergoing second SCR at the Department of Gynecologic Oncology at Fudan University Shanghai Cancer Center (FUSCC) between January 1995 and May 2012 were identified from a search of FUSCC Endometrial Cancer database. Patients with nonepithelial tumors (eg. sarcoma) were excluded. Recurrence was defined as a regrowth of tumor at least 3 months after the completion of primary therapy.

Patient data were abstracted retrospectively from inpatient and outpatient medical records, including clinical, surgery, and pathology reports of both primary and secondary surgeries. All patients were restaged by the International Federation of Gynecology and Obstetrics (FIGO) stage 2009 [11]. Progression free interval (PFI) was defined as the time from primary surgery to the diagnosis of recurrence. The level of cytoreduction for recurrent endometrial cancer was defined as: R0, complete resection with no visible disease; R1, residual disease ≤1 cm; R2, residual disease >1 cm.

The recurrent sites were categorized as solitary and multiple. Patients were divided into two groups according to the detection of recurrence: symptomatic, and asyptomatic groups.

All patients were followed up at least 3 months after surgery. Two patients died 2 months after SCR, and one patients lost follow-up after hospital discharge. The median follow-up duration was 18 months (range: 2–112 months).

### Statistical analysis

The primary statistical endpoints were overall survival (OS) calculated from the date of SCR to the date of death or last follow-up and progression-free survival (PFS) from the date of SCR to the date of progression of disease. The chi-square test was used for discrete and binomial data. Stepwise logistic regression was used to analyze the correlations between clinico-pathological variables and SCR outcome. Survival curves were estimated by Kaplan–Meier method and compared using the log-rank test. Multivariate analysis was performed by Cox proportional hazards regression model. *P* < 0.05 was set to be significant. All statistical analyses were performed using SPSS software (version 11.0).

## Results

### Patient characteristics

Total 75 patients with recurrent endometrial cancer who underwent surgery during the study period were identified. Clinico-pathological characteristics were summarized in Table 1. Median age at the first surgery was 55 years old (range: 31–75). At the first surgery, 53 (70.7%) patients had stage I disease (FIGO 2009), 5 (6.7%) had stage II, 13 (17.3%) had stage III, and 4 (5.3%) had stage IV disease. Sixty-five (85.7%) patients were diagnosed with endometrioid adenocarcinoma, and 10 (13.3%) patients with non-endometrioid adenocarcinoma, including 6 papillary serous cancer, 3 clear cell cancer, and 1 squamous cell cancer.

**Table 1:** Clinicopathological characteristics of patients and univariate analysis for OS after SCR

	**Cases**	**Deaths**	**Median OS (months)**	**UV**^**a **^**analysis (*P *** **value)**
**Age at second surgery**				**0.549**
≤**56 years**	38	15	60	
**>56 years**	37	15	39	
**ECOG performance status at first surgery**				**0.352**
**0**	6	1	48.00	
**1**	62	25	62.124	
**2**	7	4	34.00	
**Stage at first surgery (FIGO 2009)**				**0.760**
**Stage I-II**	58	22	65.675	
**Stage III-IV**	17	8	45.159	
**Pathological subtype**				**0.180**
**Endometrioid**	65	28	57.753	
**Nonendometrioid**	10	2	52.10	
**Histological grade**				
**I**	14	4	67.921	**0.068**
**II**	39	15	68.006	0.040*
**III**	22	11	44.819	**Reference**
**Postoperative therapy after first surgery**				**0.309**
**Chemotherapy**	33	14	53.51	
**Radiotherapy**	5	2	36	
**CT+RT**	6	1	72	
**Hormonal therapy**	4	1	114	
**No**	27	12	36.196	
**Symptoms at recurrence**				**0.723**
**Nonsymptomatic**	30	13	54	
**Symptomatic**	45	17	65	
**CA125 at recurrence**				**0.108**
≤ **35 U/ml**	25	7	69.650	
**>35 U/ml**	35	18	35.733	
**Not available**	15	5	77.548	
**Recurrence detection of nonsymptomatic**				**0.134**
**Imaging or CA-125**	17	8	44.83	
**Physical examination**	13	4	96	
**PFI**				0.032*
≤**18 months**	40	19	34.963	
**>18 months**	35	11	76.078	
**Largest size of recurrence**				0.008*
≤** 6 cm**	36	10	79.027	
**> 6 cm**	39	20	35.918	
**Multiplicity of recurrence**				**0.144**
**Solitary**	35	12	73.219	
**Multiple**	40	18	37.295	
**Site of recurrence**				**0.056**
**Pelvis**^**b **^**and/or introabdomonal**	63	23	66.186	
**Retroperitoneal**	12	7	30.380	
**Ascites**				0.048*
**None**	67	26	64.678	
**Yes**	8	4	17.171	
**ECOG performance status before SCR**				**0.311**
**0**	30	13	53.28	
**1**	26	9	109.31	
**2**	14	5	72.0	
**3**	5	3	14.4	
**Residual disease after SCR**				
**None**	43	12	76.462	0.000*
**0.1~1 cm**	15	6	43.825	0.001*
**1~2 cm**	6	4	30.833	**0.207**
**>2 cm**	11	8	12.778	**Reference**
**Postoperative therapy after SCR**				
**Chemotherapy**	48	22	40.192	**Reference**
**Radiotherapy**	6	1	114.0	0.045*
**CT+RT**	8	3	42.0	**0.764**
**No**	13	4	72.0	**0.615**

*Note.* a, UV, univariate; b, pelvis: including inguinal and vaginal; CT: chemotherapy; RT: radiotherapy; *P <0.05.

At the first surgery, ECOG scores were no more than 2, including 0 in 6 patients, 1 in 62 patients, and 2 in 7 patients. Twenty-seven (36.0%) patients received total hysterectomy with bilateral salpingoopherectomy (TH/BSO), 10 (13.3%) received radical hysterectomy with bilateral salpingoopherectomy (RH/BSO), 12 (16.0%) received TH/BSO and pelvic lymphadenectomy (PL), 15 (20.0%) received RH/BSO/PL, 4 (5.3%) received TH/BSO/PL and para-aortic lymphadenectomy, 7 (9.3%) received TH/BSO and cytoreduction surgery. For patients requiring primary cytoreductive surgery, an optimal resection was obtained in all patients. After the first surgery, 48 (64%) patients received adjuvant therapy, including 33 (44.0%) patients with chemotherapy, 5 (6.7%) with radiotherapy, 6 (8.0%) with both chemotherapy and radiotherapy, and 4 (5.3%) with hormonal therapy.

Median PFI was 18 months (range: 3–372). Median age at recurrence was 56 years old (range: 33–76). In recurrence, 30 patients were diagnosed without symptoms at routine follow-up, and 45 patients with various symptoms, including vaginal bleeding or discharge (17 cases), abdominal pain (16 cases), inguinal mass (3 cases), low-degree fever (3 cases), constipation (3 cases), diarrhea (1 case), hemoptysis (1 case), and lower limb edema (1 case).

Before SCR, 24 cases were treated with chemotherapy, 4 cased with both chemotherapy and radiotherapy, and 1 case with radiotherapy. The most common regimens were paclitaxel plus platinum, or platinum plus doxorubicin.

### Salvage cytoreductive surgery

Characteristics of recurrence and SCR were summarized in Table 2. ECOG scores before SCR were no more than 3, including 0 in 30 patients, 1 in 25 patients, 2 in 15 patients, and 3 in 5 patients. During SCR, median largest size of recurrence disease was 6 cm (range: 1–25). Recurrence was found solitary in 35 (46.7%) patients and multiple in 40 (53.3%) patients. Ascites were found in 8 (10.5%) patients with recurrence, with median ascites volume 450 ml (range: 200–2400 ml). After SCR, 58 (77.3%) patients achieved optimal cytoreduction (residual disease ≤1 cm). Nine (12.0%) patients developed perioperative complications, and no patient died during perioperative period.

**Table 2:** Characteristics of recurrence and second debulking surgery

	**Number of patients (%)**
Largest size of recurrence (cm)	
Median (range)	6 (1-25)
Sites of recurrence	
Central pelvic-vaginal	6 (8.0)
Pelvic	25 (33.3)
Intro-abdorminal alone	3 (4.0)
Retroperitoneal alone	8 (10.7)
Pelvic and intra-abdominal	17 (22.7)
Pelvic and retroperitoneal	3 (4.0)
Intra-abdominal and retroperitoneal	1 (1.3)
Vaginal	5 (6.7)
Inguinal	5 (6.7)
Abdominal wall	1 (1.3)
Lung	1 (1.3)
Ascites	8 (10.5)
Multiplicity of recurrence	
Solitary	35 (46.7)
Multiple	40 (53.3)
Surgical procedures	
Tumor mass resection	36 (51.4)
Pelvic lymph node resection	12 (16.0)
Para-aortic lymph node resection	10 (13.3)
Inguinal lymph node resection	4 (5.3)
Omentectomy	17 (22.7)
Appendectomy	9 (12.0)
Mesenterectomy	5 (6.7)
Large-bowel resection	19 (25.0)
Small-bowel resection	6 (8.0)
Colostomy	6 (8.0)
Patial abdominal wall resection	3 (4.0)
Upper vaginectomy	18 (24.0)
Vulvectomy	1 (1.3)
Partial urethrectomy	1 (1.3)
Partial cystectomy and ureterectomy	2 (2.7)
Ureteral stents	6 (8.0)
Partial gastrectomy	1 (1.3)
Radical pulmonary lobe resection	1 (1.3)
Biopsy alone	4 (5.3)
Operative time (minutes)	
Median (range)	150 (30-430)
Blood transfusion	42 (56.0)
Median (range, blood unit)	2 (1-13)
Median hospitalized day	20 (6-130)
Residual disease (cm)	
None	43 (57.3)
0.1~1	15 (20.0)
1~2	6 (8.0)
>2	11 (14.7)
Complications	9 (12.0)
Iliac artery injury	1 (1.33)
Ureteral injury	2 (2.67)
Acute renal failure	1 (1.33)
Urethrovaginal fistula	1 (1.33)
Bowel obstruction	1 (1.33)
Deep vein thrombosis	1 (1.33)
Drug allergy	1 (1.33)
Hydronephrosis	1 (1.33)

After SCR, 48 (64.0%) patients received salvage chemotherapy. The main regimen used was platinum plus taxol (in 29 patients). Six (8.0%) patients received radiotherapy, and 8 (10.7%) patients received both chemotherapy and radiotherapy. Twenty (26.7%) patients received hormonal therapy after SCR (5 alone, 15 combined with chemotherapy and/or radiotherapy).

### Survival analysis

During follow-up, 47 patients (62.7%) had disease progression. The median progression-free survival (PFS) was 9 months. The median survival was 18 months (range: 2–112 months). During follow-up, 30 (40.0%) patients died of disease, 14 (18.7%) were alive with disease, and 31 (41.3%) were alive without disease.

**Figure 1:**
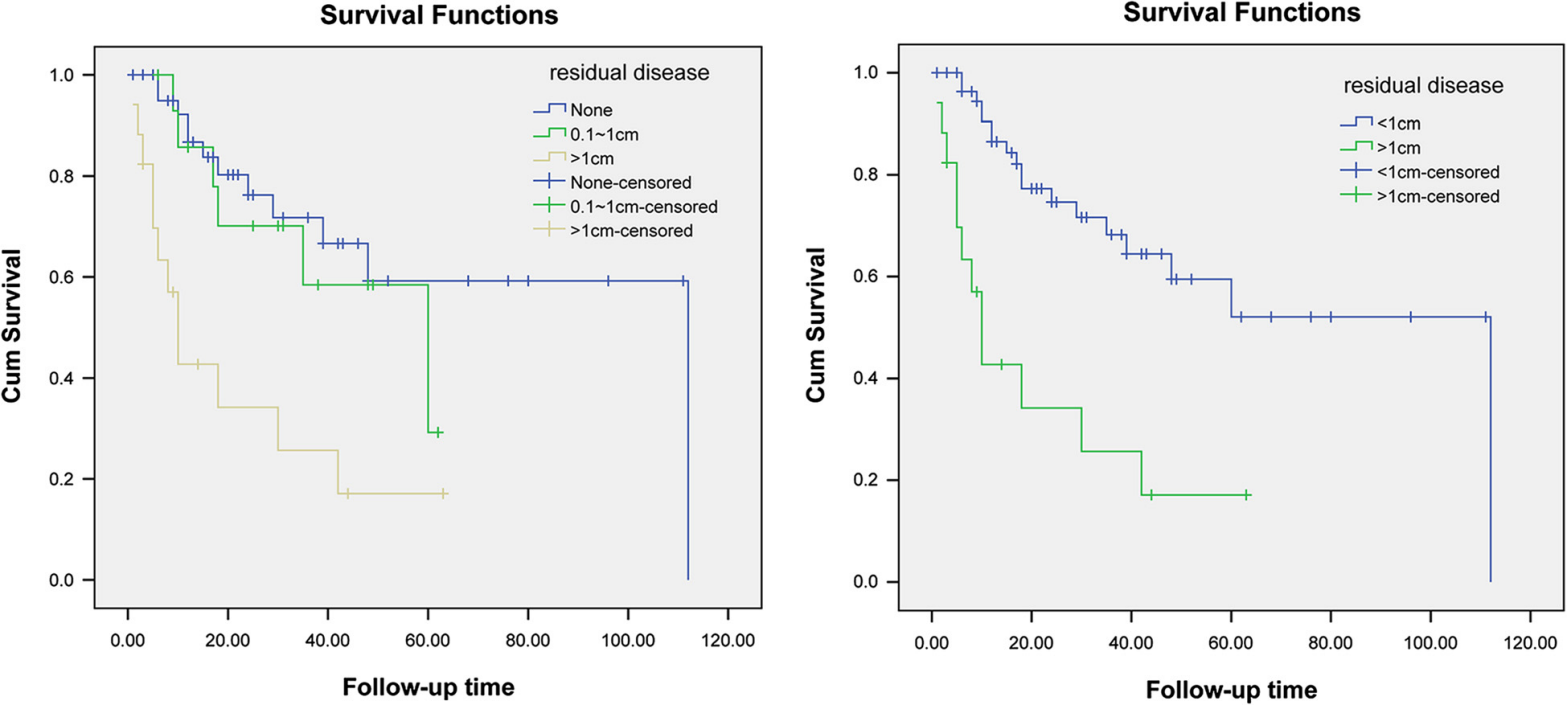
Survival curve of residual disease after second debulking surgery.

Univariate analysis showed that the residual disease of SCR, grade, PFI, the largest size of the recurrent tumors, the multiplicity of the recurrent tumors, postoperative therapy after SCR, and ascites were associated with the post-recurrent survival (*P* < 0.05, Table 1). The median survival of patients with R0, R1, and R2 were 76.5, 43.8, and 21.8 months, respectively. The differences for stratified comparisons between the outcomes of R0, R1, and R2 were significant (R0 vs. R2: χ^2^ = 15.55, *P* = 0.00, R1 vs. R2: χ^2^ = 5.45, *P* = 0.02) (Figure 1). In addition, the differences for stratified comparisons between the outcomes of residual disease of ≤1 cm and >1 cm (χ^2^ = 15.55, *P* = 0.00) were significant. However, there was no significant difference between residual disease of none and 0.1 ~ 1 cm (χ^2^ = 0.465, *P* = 0.495), and there was no significant difference between residual disease of 1–2 cm and >2 cm (χ^2^ = 1.592, *P* = 0.207). Therefore, residual disease of ≤1 cm was considered optimal cytoreduction and analyzed in multivariate analyses. The asymptomatic patients with recurrence detected by imaging or CA-125 measurements tended to have shorter survival than patients with recurrence detected by physical examination (44.83 vs. 96 months), but the difference was not significant (P = 0.134). All variables with *P* < 0.01 in univariate analysis were analyzed by multivariate analysis, and the results showed that residual disease after SCR and grade were predictive factors for survival after SCR (*P* = 0.001 and *P* = 0.012, respectively).

### Factors associated with the outcomes of SCR

After SCR, R0 resection was achieved in 43 (57.3%) patients, R1 in 15 (20.0%) patients, and R2 in 17 (22.7%) patients.

In univariate analysis, the age at recurrence, the size of the largest recurrent tumors, the site of recurrence (retroperitoneal or not), and multiplicity of recurrence were associated with optimal cytoreduction (R0 and R1) (*P* = 0.013, 0.022, 0.086, and 0.03, respectively). Multivariate analysis showed that the age at recurrence, the size of the largest recurrent tumors, and multiplicity of recurrence were associated with the optimal cytoreduction (*P* = 0.046, 0.028, and 0.044, respectively) (Table 3). Stage at primary surgery, grade, symptoms at recurrence, pathological subtype, PFI, and ascites at recurrence were not predictors of SCR outcomes.

**Table 3:** Logistic regression for clinico-pathologic variables associated with residual disease at second debulking surgery

	**UV regression**	**MV regression**		
	***P *** **value**	***P *** **value**	**Exp (B)**	**95.0% CI**
Age at second surgery (>56 years)	0.013*	0.046*	3.841	1.026-14.381
Sites of recurrence (multiple)	0.030*	0.044*	4.061	1.039-15.868
Largest size of recurrence (> 6 cm)	0.022	0.028*	4.611	1.180-18.016
Site of recurrence (retroperitoneal)	0.086	0.156	3.027	0.656-13.955

*Note.* UV, univariate; MV, multivariate; 95%CI, 95% confidence interval; *P <0.05.

## Discussion

The treatment for recurrent endometrial cancer varies depending on the recurrent location, the extent of disease, and prior therapy used. For patients with a localized vaginal recurrence and no previous irradiation, radiation therapy could provide a long-term pelvic control and 5-year survival rate of 31-53% [12,13]. The majority of patients with recurrence are treated with palliative chemotherapy, and/or hormonal therapy. The commonly used chemotherapy included cisplatin, carboplatin, doxorubicin, paclitaxel, and topotecan, with overall response rate ranging from 20% to 37% [14-18]. The response rate of hormonal therapy with progestational agents, anti-estrogens, and gonadotropins-releasing hormone analogs are generally low, ranging from 9% to 16% [19-21]. Surgical resection for recurrent endometrial cancer has traditionally been limited to a selected group of patients presenting with a central pelvic recurrence within a previously irradiated pelvic field. However, only few patients are candidates for this approach [5,6].

To date, only 4 nonrandomized, retrospective studies have investigated the outcomes of cytoreductive surgery for recurrent endometrial cancer [7-10] (Table 4). A meta-analysis on these 4 studies was performed to determine the use of cytoreductive surgery [22]. Patients undergoing optimal surgical cytoreduction (ranging from <2 cm to no gross residual disease) had an overall survival advantage. However, the subset with no gross residual disease (complete surgical cytoreduction) was associated with improved survival [22]. In this study, we confirmed the survival benefit of patients with optimal cytoreduction. In multivariate analysis, residual disease after SCR and grade were found to be the predictive factors for survival after SCR. Especially, residual disease was the strongest survival determinant for recurrent endometrial cancer (*P* = 0.001).

**Table 4:** Summary of studies on cytoreductive surgery of recurrent endometrial cancer

	**Case**	**PFI (m)**	**Surgery patients**	**OS (m)**	**Optimal definition (cm)**	**Optimal cytoreduction (%)**	**Optimal median OS (m)**	**Suboptimal median OS (m)**
Scarabelli [7]	20	16.9	20	NA	No gross	65 (13/20)	12	Undefined
Campagnutta [8]	75	13	75	19	<=1	74.7 (56/75)	53	9
Bristow [9]	61	18.5	35	28	No grossly visible	65.7 (23/35)	39	13.5
		/	26	13	/	/	/	/
Awtrey [10]	27	20	27	35	<=2	67 (18/27)	43	10
This study	75	18	75	18	<=1	77.3(58/75)	72	22

*Note.* NA, not assessable. PFI, progression free interval. OS, overall survival.

In previous studies, the definition of optimal cytoreduction was ≤1 cm or ≤2 cm. In this study, we found that patients with ≤1 cm residual disease had significant survival benefit from cytoreductive surgery, compared with the patients with >1 cm residual disease (χ2 = 15.55, P = 0.00), while there was no difference in survival between patients with none disease and 0.1-1 cm (χ2 = 0.465, P = 0.495), and between 1–2 cm and >2 cm (χ2 = 1.592, P = 0.207). Therefore, residual disease of ≤1 cm was considered optimal cytoreduction and pooled together for analysis. These data suggest that the definition of optimal cytoreduction might be <1 cm, but further studies employing larger samples are needed to confirm this definition.

On the other hand, careful selection of the patients is important due to the complications of SCR. In this study we attempted to predict the optimal cytoreduction. The factors associated with optimal cytoreduction in recurrent endometrial cancer were identified by logistic regression analysis. In univariate analysis, the age, PFI, the size of the largest recurrent tumors, the site of recurrence, and multiplicity of recurrence were associated with optimal cytoreduction, which indicates that patients with younger age, longer PFI, smaller tumor size, and recurrent site not in retroperitoneal, and single recurrence are more likely to achieve optimal cytoreduction. However, in multivariate analysis, only the age, the size of the largest recurrent tumors, and multiplicity of recurrence were associated with the optimal cytoreduction. Therefore, patients with young age (<56 years old), small tumor size (<6 cm) and solitary recurrence are more likely to achieve optimal cytoreduction and gain benefit.

After SCR, the patients who received radiotherapy after SCR showed better OS than patients with chemotherapy (p = 0.045). The reason may be due to the fact that more than half of the patients (48 cases, 64%) received chemotherapy with widely disseminated tumor, and only 6 patients received radiotherapy with the disease localized to vagina or vulva. However, adjuvant therapy did not show significance in multivariate analysis. In a previous study the median survival of symptomatic patients was longer than that of asymptomatic patients (27 vs. 12 months); although the difference was not statistically significant [23]. Consistent with this, we found that the median survival of symptomatic patients was longer than that of asymptomatic patients (65 vs. 54 months), but the difference was not statistically significant (P = 0.723). In addition, we found that patients with recurrence detected by imaging or CA-125 measurements tended to have shorter survival than patients with recurrence detected by physical examination (44.83 vs. 96 months), but the difference was not significant (P = 0.134). Therefore, early detection of recurrence by imaging and CA-125 measurements can not improve prognosis. Routine follow-up for these patients is necessary to detect recurrence by physical examination.

A dramatic survival advantage of SCR was observed in carefully selected patients. However, due to the radical procedures involved, the incidence of major surgical complications and deaths should be concerned. According to the literatures, the rate of complications varied from 25% to 62.7% (Table 5) [7-10]. In our study, total 9 patients (12.0%) developed perioperative complications, including 1 iliac artery injury, 2 ureteral injury, 1 acute renal failure, 1 urethrovaginal fistula, 1 bowel obstruction, 1 deep vein thrombosis, 1 hydronephrosis, and 1 drug allergy, while 42 patients (56%) received blood transfusion during surgery. No patient died during perioperative period. The rate of complication in our study was similar to previous studies with blood transfusion included. Therefore, SCR was safe and acceptable in general, but the incidence of severe complications should be concerned. One study reported two (10%) perioperative deaths after 21 and 30 days, one of them had a history of cerebrovascular and hypertensive disease and developed postoperative hemorrage which required reintervention within 18 h of surgery [7]. In another study, one patient with diffuse carcinosis died after surgery, whereas five other patients (three with diffuse carcinosis) died in the hospital postoperatively [8]. In this study, four patients with diffuse carcinosis died postoperatively. These data suggest that in patient with carcinosis it is not suitable to perform SCR, and adequate preoperative evaluation is necessary to reduce the rate of perioperative death.

**Table 5:** Summary of complications of SCR

	**Cases**	**Complications**	**Patients with BT**	**BT unit**	**Perioperative deaths**
Scarabelli [7]	20	3 (15%)	Not known	1.8 (0–5)	2 (10%)
Campagnutta [8]	75	23 (30.7%)	Not known	1.3 (0–8)	1 (1.3%)
Bristow [9]	35	13 (37.1%)	10 (28.6%)	3 (1-6)	None
Awtrey [10]	27	17 (62.7%)	9 (33.3%)	Not known	None
This study	75	9 (12%)	42 (56%)	2 (1-13)	None

*Note.* BT, Blood transfusion.

In this study, optimal median OS was significantly longer than that reported in previous studies, but there is no much difference in OS (Table 4). Several reasons may account for it: first, the definition of optimal cytoreduction varied in different studies (ranging from <2 cm to no gross residual disease), which influences OS with optimal cytoreduction. Second, the selection of patients is different among different studies. For example, 9 patients who had resection of non-abdominal disease and 13 patients who underwent a pelvic exenterative procedure were excluded in one study [10]. In this study, 4 patients receiving inguinal lymph node resection were included, who had much better OS than patients with abdominal recurrence if optimal cytoreduction was achieved.

There are several limitations of our study. First, the number of patients enrolled in this study is small, due to the infrequency of recurrent endometrial cancer. Therefore, the selection criteria for SCR need to be confirmed in further study. Second, there is a potential for selection bias because our study is retrospective. Prospective study is necessary to identify the appropriate candidates for SCR. Third, we failed to collect the data of the patients who did not undergo cytoreduction and compare them to the patients enrolled in this study, which would strengthen our conclusion that patients with recurrent endometrial cancer would benefit from SCR.

## Conclusions

Based on our data, we get the following conclusions. First, patients with residual disease less than 1 cm could benefit from optimal SCR with prolonged overall survival. Second, patients should be carefully selected before the surgery, as patients with younger age, tumor size less than 6 cm, and single recurrence are more likely to achieve satisfied cytoreduction. Third, complications of SCR are not common but should be considered.

## Competing interests

The authors declare that they have no competing interests.

## Authors’ contributions

YR and BS performed the study. DS performed statistical analysis. HW conceived the study. All authors read and approved the final manuscript.

## Pre-publication history

The pre-publication history for this paper can be accessed here:

http://www.biomedcentral.com/1471-2407/14/135/prepub
